# Progress in Clinical Neurosciences, Cognitive Neurosciences, Clinical Psychology, Neurotechnology and Brain Mapping in Malaysia

**DOI:** 10.21315/mjms2021.28.2.1

**Published:** 2021-04-21

**Authors:** Jafri Malin Abdullah

**Affiliations:** Malaysian Journal of Medical Sciences, Universiti Sains Malaysia, Kubang Kerian, Kelantan, Malaysia

**Keywords:** neurotechnology, brain mapping, manpower, clinical psychologist, cognitive neuroscientist, neurosciences, precision medicine, Malaysia

## Abstract

Last year, there was an increase in the amount of manpower in Malaysia, especially in terms of the numbers of neurosurgeons, cognitive neuroscientists and clinical psychologists. One way to increase the number of cognitive neurotechnologists in the country in 2021 is to allow neuroscientists to register as neurotechnologists with the Malaysian Board of Technologists (MBOT). The Malaysian Brain Mapping project has risen from its humble beginnings as an initiative of the Universiti Sains Malaysia Brain Mapping Group in 2017. There is currently a proposal for its entry into the national arena via the Precision Medicine Initiative with the Academy Science Malaysia, the Ministry of Science, Technology and Innovation, Ministry of Higher Education and Ministry of Health. The current Malaysian Government’s Science, Technology, Innovation and Economy (STIE) plan was launched in 2020, leading to the establishment of neurotechnology as one of 10 STIE drivers.

## Introduction

On 14 July 2020, the Prime Minister of Malaysia, Tan Sri Muhyiddin Yassin, chaired the National Science Council with the Ministry of Science, Technology and Innovation’s Minister Khairy Jamaluddin, Deputy Minister Ahmad Amzad Hasim and Secretary-General Datuk Ir Dr Siti Hamisah Tapsir and launched 10 Science, Technology, Innovation and Economy (STIE) drivers in accordance with the Malaysian National Policy on Science, Technology and Innovation (DSTIN) 2021–2030. One of the STIE drivers was neurotechnology (1).

Neurotechnology took many years to be recognised in Malaysia. In 2017, the Academy of Science Malaysia prepared a report entitled *Science & Technology Foresight Malaysia 2050: Emerging Science, Engineering & Technology (ESET) Study.* Then, in December 2020, the 10–10 MySTIE framework, which trailblazed a path for prosperity, societal well-being and global competitiveness, was published and officiated by Minister Jamaluddin with the New Science Policy: DSTIN 2030 (2, 3).

On 15 December 2020, Bank Negara Malaysia established a RM1 billion High Tech Facility-National Investment Aspiration (HTFNIA) as part of its efforts to provide additional assistance for small medium enterprises (SMEs) affected by COVID-19. SME project participants in key government programmes involved in research, development and innovation for critical technologies identified under national blueprints from IR 4.0-related technologies, green technology and biotechnology to ensure continuity and the completion of existing projects. These technologies included blockchain, artificial intelligence, big data analytics, internet of things, addictive manufacturing (3D/4D/5D/6D printing), cybersecurity, system integrators, augmented reality, advanced materials, drones and manufacturing systems as well as bioscience technology and neurotechnology (4).

## Moving a New Generation Forward During the COVID-19 Pandemic in Malaysia

**Figure 1:**
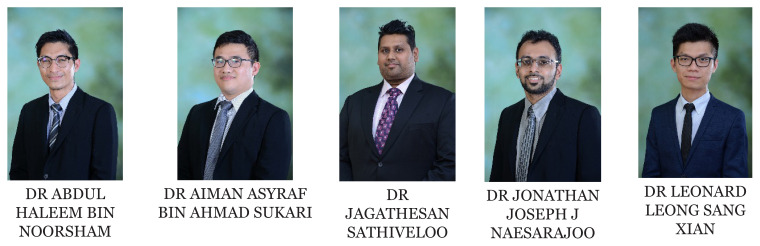

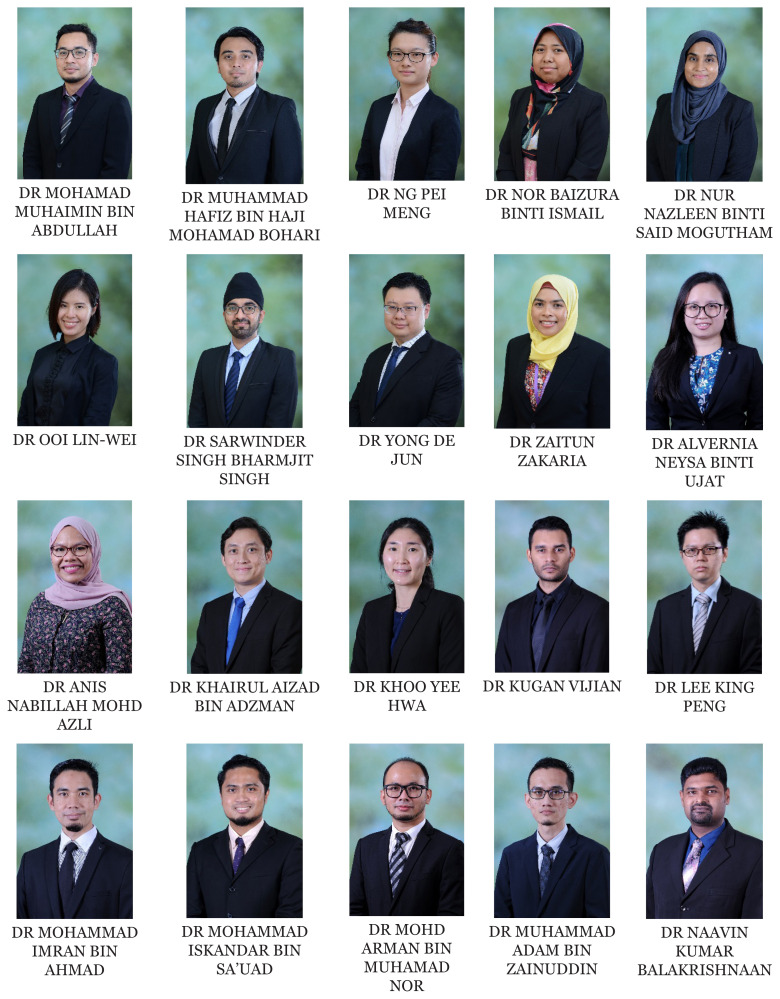

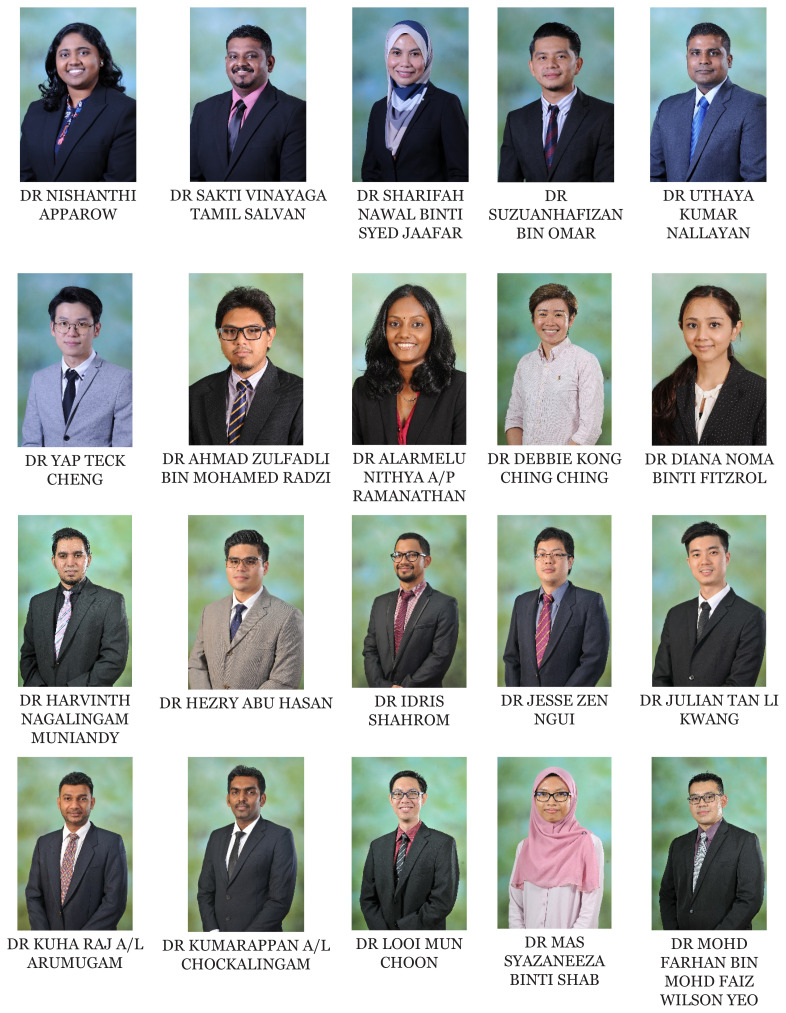

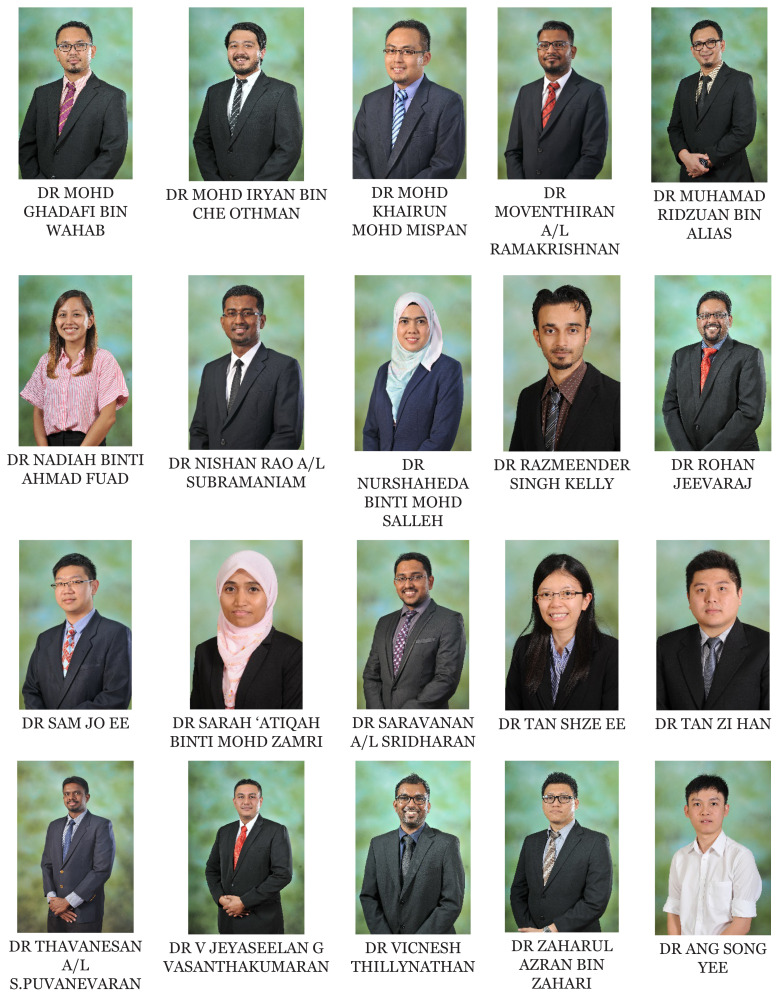

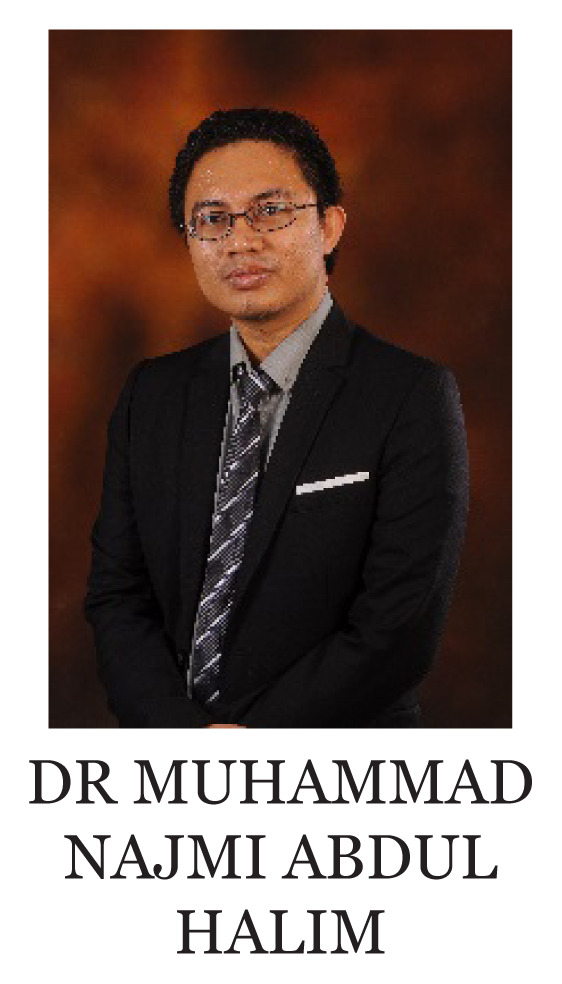
Masters of Surgery (Neurosurgery) residents from May 2019 till December 2020

**Figure 2:**
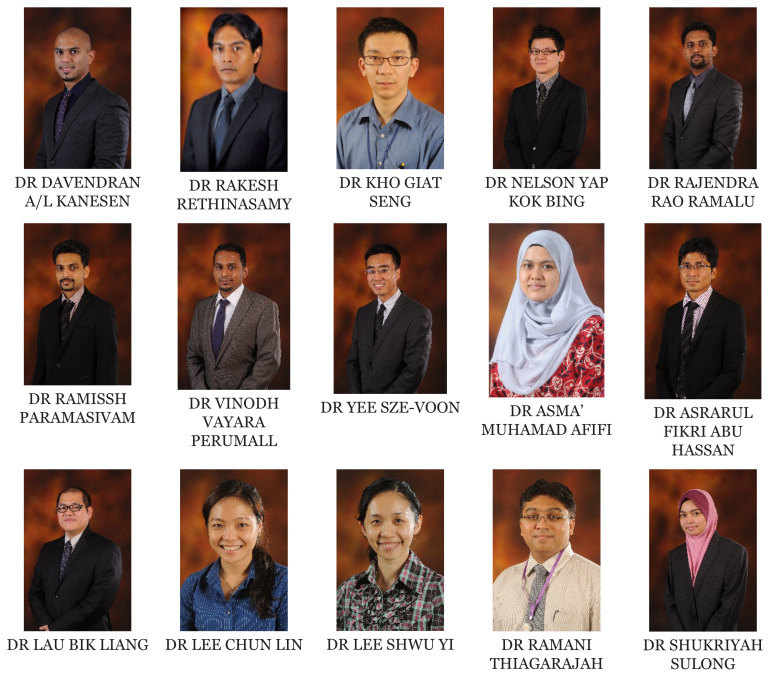

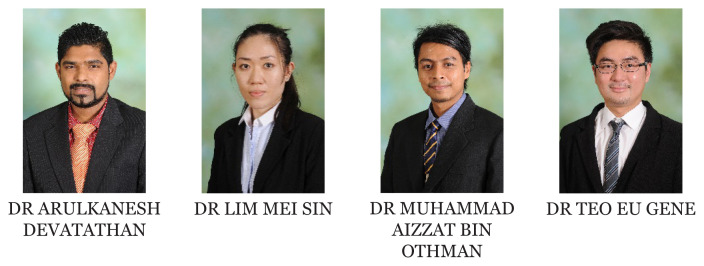
Neurosurgeons who have graduated from postgraduate neurosurgical programme from 2019

**Figure 3:**
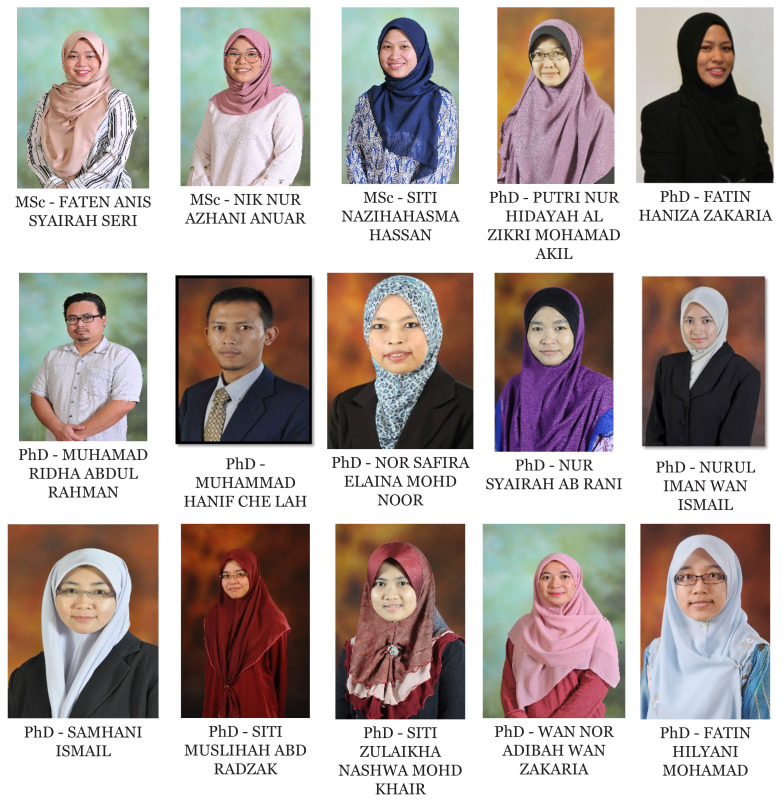

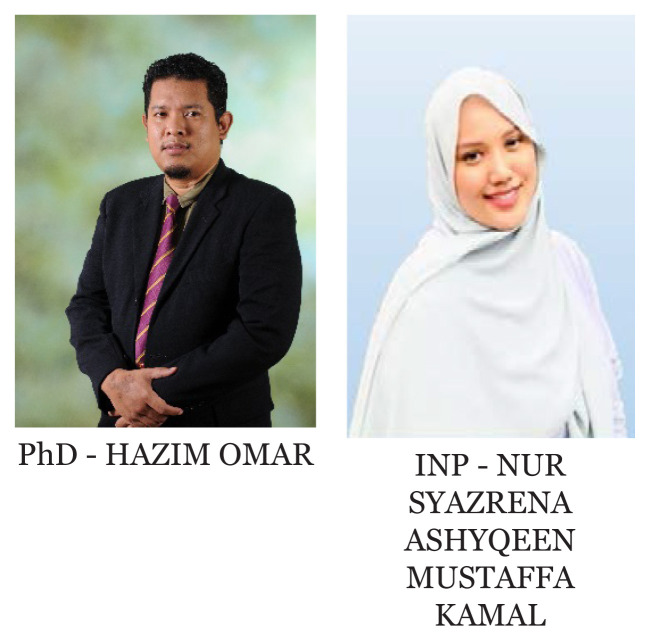
Masters by Pure Research/Mixed Mode and PhD by Pure Research from May 2019

**Figure 4:**
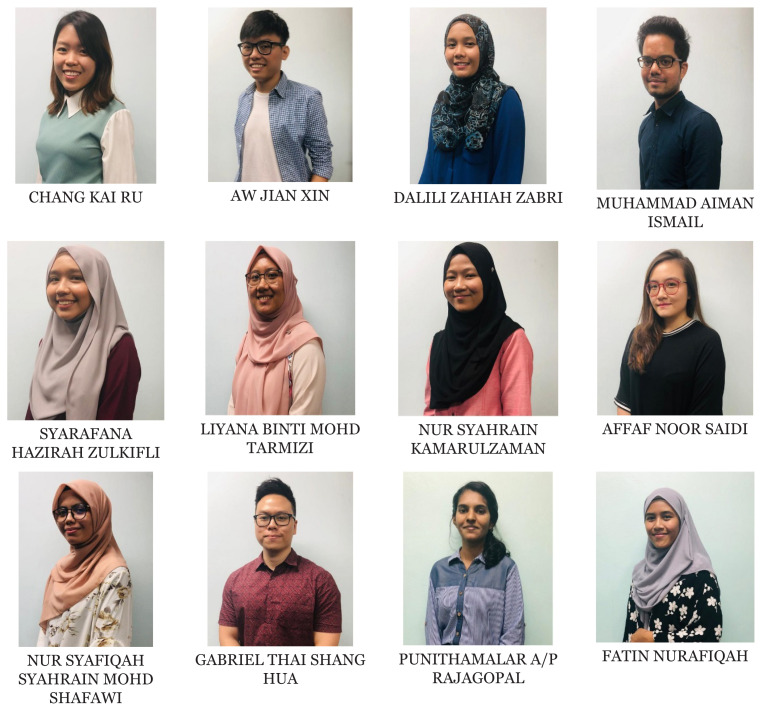

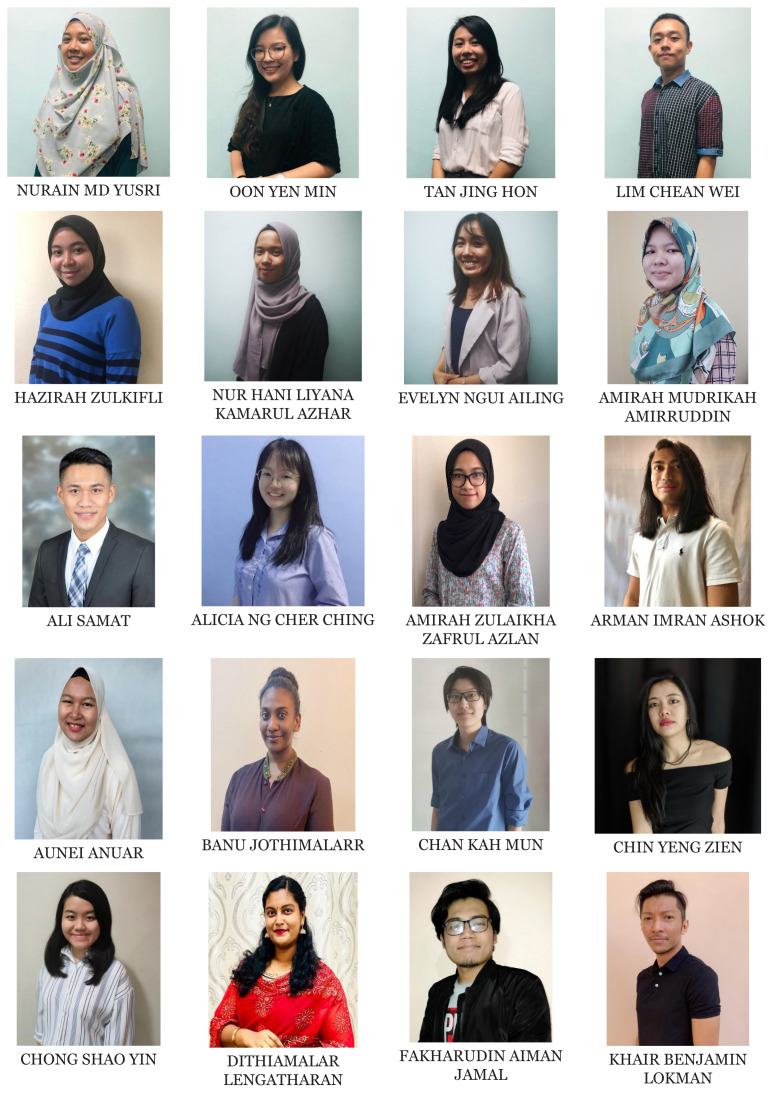

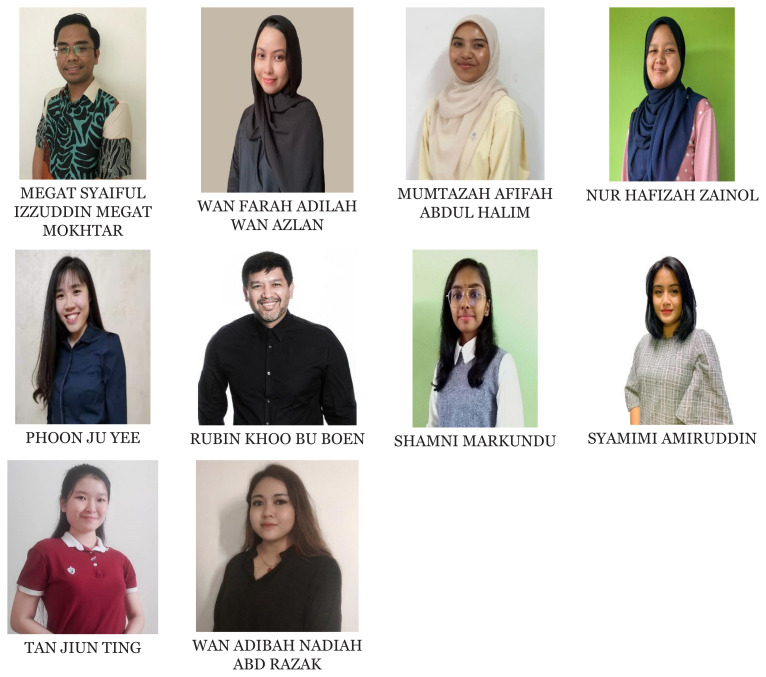
Second and third batch of Clinical Psychology students USM-UPSI

**Figure 5:**
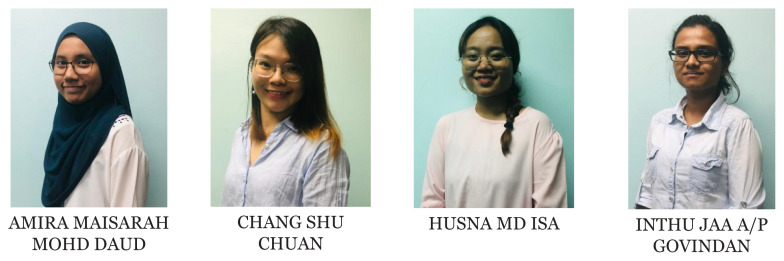

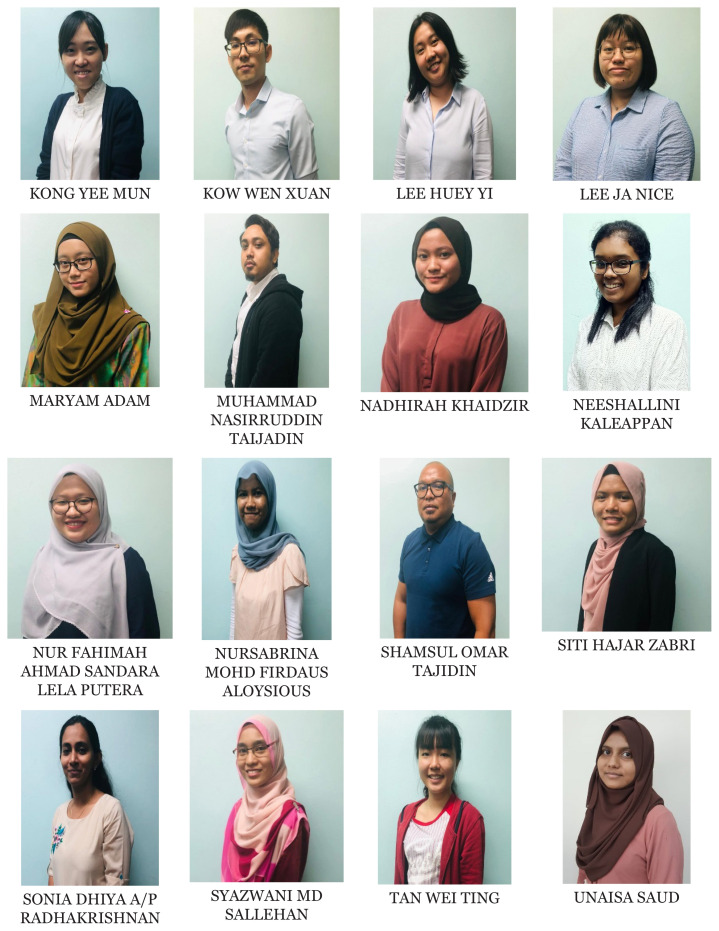

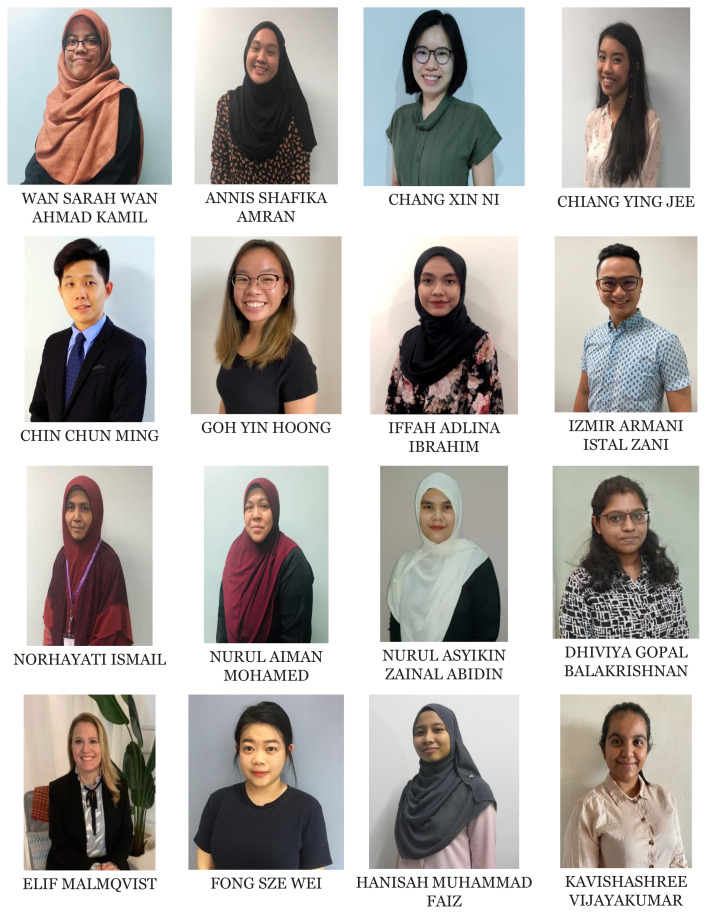

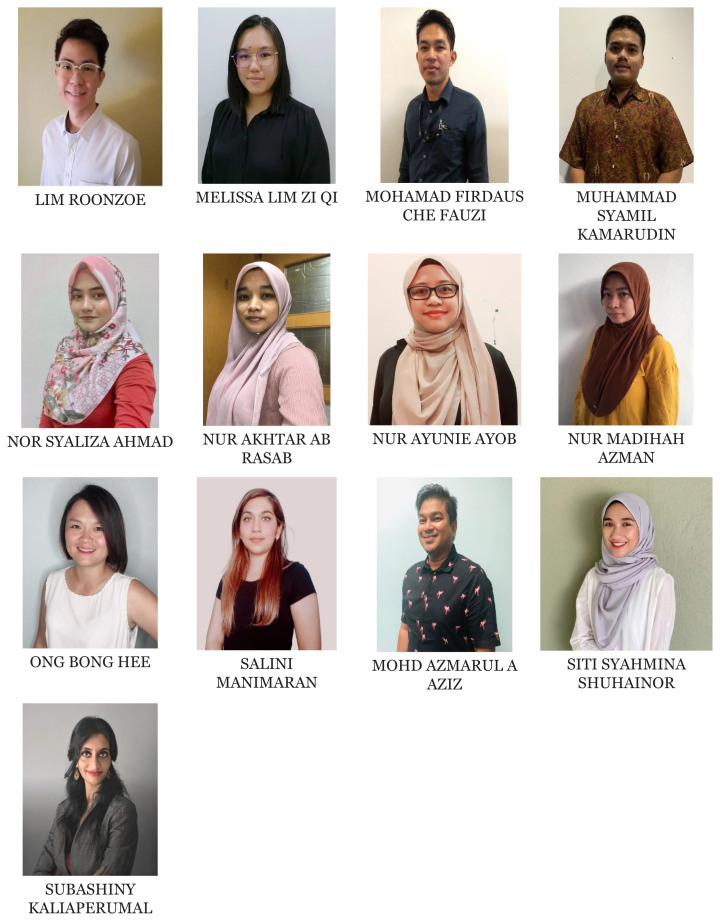
Third, fourth and fifth batch of Masters of Cognitive Neurosciences USM offered at Postgraduate Institute @Kuala Lumpur

Figures 1–5 show the current batches of manpower being trained after our last report a year ago (5, 6). The percentages of our neuroscience and psychology graduates being hired up to 2021 from the end of 2019 were: 100% for Masters of Surgery (Neurosurgery) and Advanced Masters of Medicine (Neurology), 50% for PhDs/Doctorate, 55%–75% for the Masters of Cognitive Neurosciences and the Integrated Programme, and 44% for the Clinical Psychology graduates (7).

The Malaysian Brain Mapping project that uses various neurotechnologies (electroencephalography, functional magnetic resonance imaging, event related potential, eye tracking, magnetoencephalography, deep brain microrecording, near infrared spectroscopy) has risen from its humble beginnings as an initiative of the Universiti Sains Malaysia Brain Mapping Group in 2017. There is currently a proposal for its entry into the national arena via the Precision Medicine Initiative with the Academy Science Malaysia, the Ministry of Science, Technology and Innovation, and the Ministry of Health in a recent meeting with the Academy of Science Malaysia in early 2021. This is a Malaysia’s parallel initiative of the successful Cuban Brain Mapping Project which was published recently (8).

Thus, Malaysia must have a Centre of Excellence for Clinical Neuroscience, Psychiatry and Psychology services that, at least, represents the cluster of hospitals and teaching institutions with clinical neurosciences as well as psychiatry and clinical psychological services situated in the east coast of West Malaysia in the 12th Malaysia Plan that emphasises the use of neurotechnology in healthcare. It is also important to consistently build the younger generation of neuroscientists, neurologists, neurosurgeons, neurorehabilitation specialists, clinical psychologists and clinical neuropsychologists since it takes nearly 11 to 16 years to train them to address the needs of the country using neurotechnology to diagnose and cure diseases.
